# Multi-Hardware Benchmarking of Open-Source Large Language Models with Retrieval-Augmented Generation for Mitsubishi FX-Series PLC Instruction List Code Generation

**DOI:** 10.3390/s26113602

**Published:** 2026-06-05

**Authors:** Ming-Feng Yeh, Ching-Chuan Luo, Cheng-Lin Lu

**Affiliations:** 1 Department of Electrical Engineering, Lunghwa University of Science and Technology, Taoyuan 333326, Taiwan; mfyeh@mail.lhu.edu.tw (M.-F.Y.); g1122161006@gm.lhu.edu.tw (C.-L.L.); 2 Department of Electrical Engineering, Ming Chi University of Technology, New Taipei 243303, Taiwan

**Keywords:** programmable logic controller, Instruction List, Mitsubishi FX-series, large language model, retrieval-augmented generation, static syntax verification, smart manufacturing, sustainable manufacturing, industrial monitoring, on-premise AI for industrial automation

## Abstract

Smart manufacturing relies on programmable logic controllers (PLCs) that translate sensor inputs into actuator commands. Generating PLC programs in legacy textual languages such as Mitsubishi FX-series Instruction List (IL) remains an expert-only task, and IL’s deprecation in IEC 61131-3 Edition 3.0 leaves it under-represented in the corpora that train modern large language models (LLMs). We benchmark ten open-source LLMs (five vendors, 7B–122B parameters) in both LLM-only and Retrieval-Augmented Generation (RAG) configurations on a frozen 285-question dataset; the pipeline uses ChromaDB with all-MiniLM-L6-v2 embeddings and Maximal Marginal Relevance (MMR) retrieval (k=3, λ=0.5). To move beyond lexical similarity we introduce a three-tier static syntax checker (Lexical/Syntactic/Semantic) calibrated against a 93.3% ground-truth pass rate. RAG raises the syntactic pass rate by +6.7 to +61.1 percentage points across all ten models; the best configuration, qwen3.5:122b with RAG, reaches 95.8%, exceeding the ground-truth baseline. Two outliers (llama3.3:70b at +6.7 pp, gpt-oss:120b at +25.6 pp) are reported rather than excluded. The results indicate that for deprecated-but-deployed industrial languages a curated dialect corpus paired with a locally-hosted open-source LLM is more effective than scaling raw model size, supporting reproducible, on-premise industrial-monitoring and code-generation tooling for sustainable smart manufacturing.

## 1. Introduction

Smart manufacturing depends on a tightly coupled sensor–controller–actuator loop in which physical signals from limit switches, photoelectric sensors, push-button stations, encoder pulses, and process transmitters are converted into discrete control decisions. Those decisions in turn drive solenoids, contactors, motor starters, and indicator lights [1,2]. The element that closes this loop in the overwhelming majority of factory installations is the programmable logic controller (PLC). Sensor inputs (X-points), internal auxiliary relays (M-points), timer/counter blocks (T/C), data registers (D), and actuator outputs (Y-points) are wired together by a control program that the controller scans cyclically. From the artificial intelligence perspective, a PLC program is therefore a low-level, deterministic, real-time policy whose inputs are sampled sensor states and whose outputs are commanded actuator states. Producing this policy correctly is mission critical: a syntactic error blocks compilation, and a semantic defect reaches the plant floor as a safety or productivity hazard.

PLC programs are written in one of the textual or graphical languages standardised by IEC 61131-3 [3,4]. Among the textual languages, Instruction List (IL), with its assembly-like one-instruction-per-line syntax and explicit operand prefixes X, Y, M, T, C, D, S, K, H, remains the dominant low-level representation on Mitsubishi FX-series controllers. FX-series controllers remain widely deployed in small-to-medium automation, machine retrofits, vocational education, and Asian manufacturing. IL was deprecated in IEC 61131-3 Edition 3.0 and removed in Edition 4.0 [3], but the installed base of FX2N/FX3U/FX3UC controllers and the corresponding GX Works2 engineering tooling means IL programs continue to be written, maintained, and taught in practice.

It is reasonable to ask why an LLM-assisted workflow is worth building for a language that the standards body has formally retired. The motivation is operational rather than aspirational. Three concrete demand signals support the choice. First, the deployed footprint of FX2N/FX3U/FX3UC controllers in Asian small-to-medium manufacturing and machine-tool retrofit work is substantial, and these controllers run IL programs that need to be debugged, extended, and adapted on an ongoing basis; replacing them with greenfield IEC 61131-3 hardware is rarely cost-justified for one-off modifications, so the IL programs themselves have to be maintained in place. Second, the engineering manuals shipped with GX Works2 continue to document the IL mnemonic set in full, and IL is still taught in regional vocational and technical-college curricula as an entry-level industrial controller language; new engineers therefore still encounter it. Third, the audience the present pipeline targets—maintenance engineers reading or extending an existing FX-series program, and educators preparing IL exercises against a known dataset—is precisely the audience that benefits most from a generation-and-static-check loop, because the labor cost of writing and re-verifying IL by hand is high relative to the typical length of a program. We do not propose IL as the preferred language for new IEC 61131-3 projects; Structured Text and Ladder Diagram remain the recommended choices for greenfield deployments. The contribution here is to the modernisation-and-maintenance setting, not the next-generation setting.

Large language models (LLMs) have driven a surge in automated code generation [5,6,7,8], but the overwhelming majority of empirical work targets web programming languages (e.g., Python, JavaScript, and Java) on benchmarks such as HumanEval and MBPP. Industrial control languages—especially deprecated ones like IL—are under-represented in pre-training corpora; consequently, off-the-shelf LLMs frequently fabricate non-existent mnemonics, mismatch operand counts, or output prose pseudocode in place of compilable instructions. Two recent works specifically target PLC code generation: LLM4PLC [9] pairs an LLM with grammar checkers, compilers, and an SMV verifier for IEC 61131-3 Structured Text (ST) and Ladder Diagram (LD); Agents4PLC [10] introduces a multi-agent closed-loop generation-and-verification benchmark for ST. Koziolek et al. [11] were among the first to combine retrieval-augmented generation (RAG) [12,13] with control-code synthesis, retrieving function blocks from the OSCAT library to ground GPT-4 outputs.

This contrast between high-resource and deprecated languages is plausibly structural rather than merely empirical. The deprecation of IL in IEC 61131-3 Edition 3.0 [3] has been accompanied by a measurable contraction of new public IL material: open-source tutorials, GitHub repositories, and Stack Overflow discussions on IL are heavily outweighed by Structured Text and Ladder Diagram content. LLMs whose pre-training corpora closed in 2023 or later are therefore likely to inherit a distribution in which IL coverage is thinner than that of Python, Java, ST, or LD; the recent code-LLM survey of Jiang et al. [8] documents this asymmetry across general code-generation evaluation. The practical consequence we hypothesise—and the conjecture this paper sets out to evaluate—is that FX-series IL falls into a strategic blind spot of LLM pre-training. This is a deliberately scoped phrase: we use it to denote the FX-IL regime our experiments cover, namely a low-resource industrial language with persistent industrial demand, and we make no claim that the same regime applies to high-resource languages or to other industrial dialects without separate empirical verification. Within this regime, the operative bottleneck is plausibly not raw model capability but access to a curated, dialect-specific reference corpus, which is precisely what retrieval-augmented generation [12,13] supplies. The experimental contribution of this paper is to test that conjecture on a uniform ten-model sweep rather than to argue it on logical grounds alone.

These prior efforts share three common limitations. First, they target ST or LD; we are not aware of a published systematic benchmark for FX-series IL, the legacy textual language with the largest deployed footprint in Asian small-to-medium manufacturing. Second, they typically evaluate one or two proprietary models (GPT-4 family) on a small problem set; the question of how open-source LLMs scale across hardware tiers (consumer GPU through workstation accelerator) is unanswered. Third, evaluation has relied predominantly on lexical similarity (BLEU and ROUGE) or embedding-based similarity, which is insufficient evidence of functional correctness: a generated program can score high on cosine similarity while still failing a static check on operand format, mnemonic spelling, or program network start.

This paper addresses the three gaps directly. The framing we evaluate is that FX-series IL sits in the strategic blind spot of LLM pre-training described above: the language has been deprecated since IEC 61131-3 Edition 3.0 [3], reducing its open-web footprint, while remaining widely deployed in Asian small-to-medium manufacturing and vocational education. Under this framing, the regime of interest is one in which a locally hosted open-source LLM augmented with a curated domain corpus may be competitive with cloud-scale general purpose models—a claim that is testable rather than assumed. The contributions of this paper are as follows:

- Multi-vendor, multi-hardware benchmark. We evaluate ten open-source LLMs—Meta llama3.1:8b and llama3.3:70b; Alibaba qwen2.5-coder at 7B/14B/32B and qwen3.5:122b; Mistral AI mistral:latest and mistral-small3.1:24b; OpenAI gpt-oss:120b; NVIDIA nemotron-3-super:120b—on three hardware tiers (RTX 3090 24GB, RTX 4090 24GB, NVIDIA DGX Spark 128GB unified memory), each tested in both LLM-only and LLM + RAG configurations on the same 285-question dataset.
- Static syntax-checker calibrated on the FX3U/FX3UC instruction set. A three-tier validator—Lexical mnemonic recognition, Syntactic operand format and operand count validation, Semantic program network and stack balance checks—supplies a static correctness signal beyond cosine, BLEU, and ROUGE. The checker, calibrated against a 93.3% ground-truth pass rate, is explicitly a lower bound on functional correctness rather than a substitute for controller-side validation (Section 6.6).
- Retrieval-depth ablation. A controlled k∈{1,3,5} ablation on qwen2.5-coder:7b quantifies the marginal value of additional retrieved exemplars on similarity, BLEU, ROUGE, and inference time.
- Honest failure-mode analysis. We report two negative findings prominently: llama3.3:70b gains only +6.7 percentage points in syntactic pass rate from RAG, versus a median of +47 pp; gpt-oss:120b gains only +25.6 pp with the lowest absolute RAG pass rate (43.9%) among the four XL models. For llama3.3:70b we also report the result of an empirical diagnostic on the per-sample CSV that re-attributes the dominant failure to prose preamble contamination rather than to the originally hypothesised output truncation (Section 6.2).

The remainder of the paper is organised as follows. Section 2 reviews LLM-based code generation, retrieval-augmented generation, and AI-assisted PLC programming. Section 3 formalises the dataset, RAG pipeline, syntax checker, and evaluation metrics. Section 4 details models and hardware. Section 5 reports the multi-model benchmark, the syntax check matrix, and the retrieval-depth ablation. Section 6 situates the findings against the sensor–actuator framing, presents the empirical failure-mode diagnostics, and lists limitations. Section 7 concludes.

## 2. Related Work

### 2.1. LLM-Based Code Generation

The Transformer architecture [5] provides the backbone for almost every modern code generation model. From the GPT-3 generation onward [6], large autoregressive language models have demonstrated few-shot programming capability, and dedicated code-tuned variants such as Codex [7], Code Llama [14], and StarCoder [15] substantially raised pass rates on HumanEval and MBPP. Open-source instruction-tuned models including Llama 3 [16], Mistral 7B [17], and the Qwen2.5/Qwen2.5-Coder family [18] have closed much of the gap to proprietary models on general code benchmarks. A recent comprehensive survey by Jiang et al. [8] catalogs roughly five years of LLM-for-code research and observes that the dominant evaluation paradigm relies on functional pass rates against unit tests. Such test harnesses exist for high-resource general purpose languages but are largely absent for industrial control languages.

This benchmark gap motivates two design choices in the present work. First, because no executable test harness exists in the public domain for FX-series IL programs (commercial GX Works toolchains are licensed and Windows only), we substitute a static analysis validator that we calibrate against the human-authored ground-truth dataset. Second, we restrict attention to open-source models that can run on locally administered hardware: industrial customers typically cannot send proprietary control logic descriptions to public cloud APIs.

### 2.2. Retrieval-Augmented Generation

Retrieval-Augmented Generation, introduced by Lewis et al. [12], augments an LLM at inference time with documents retrieved from an external corpus, decoupling factual recall from parameter memory and substantially reducing hallucination [19]. The recall component frequently uses dense embeddings and a vector index; Dense Passage Retrieval [20] demonstrated that learned dual-encoder retrievers outperform BM25, and a parallel line of work on sentence-level embeddings [21,22] has produced lightweight models suitable for indexing tens of thousands of documents on a single GPU. The recent survey by Gao et al. [13] taxonomizes the field into Naive, Advanced, and Modular RAG and emphasizes that retrieval quality, not generator capacity, is often the limiting factor for domain-specific tasks. Maximal Marginal Relevance [23] is a long-standing post-retrieval reranking criterion that trades query relevance against intra-result diversity; it is particularly relevant for code-snippet retrieval, where many candidate exemplars from the same template family would otherwise crowd out alternative patterns.

We adopt the lightweight, locally hostable end of this spectrum: ChromaDB [24] as the vector index, all-MiniLM-L6-v2 for query and document embeddings, and MMR with λ=0.5 to balance relevance against diversity. The aim is reproducibility on commodity hardware rather than maximal retrieval accuracy.

### 2.3. AI for PLC Programming

Direct application of LLMs to PLC programming has emerged only recently. LLM4PLC [9] couples an LLM with grammar checkers, compilers, and an NuSMV verifier in a user-guided iterative loop targeting IEC 61131-3 ST and LD on the OpenPLC platform; the authors report that off-the-shelf GPT-4 and LLaMa 2 produce few directly compilable programs and that the verification loop is necessary. Agents4PLC [10] extends this to a multi-agent closed-loop framework for ST that incorporates RAG, prompt engineering, and chain-of-thought, and uniquely contributes a verifiable benchmark with formal specifications and human-checked reference programs. Koziolek et al. [11] were among the first to demonstrate retrieval augmentation for ST, retrieving OSCAT function blocks via FAISS to ground GPT-4 outputs, with compile-and-simulate validation through OpenPLC. An earlier study by the same group [25] evaluated GPT-4 across 100 prompts in 10 categories on IEC 61131-3 ST, providing a foundational baseline for LLM applicability to control logic. Most recently, Kersting et al. [26] report a vendor-aware on-premise pipeline that fine tunes a small local model and retrieves Mitsubishi Electric-specific function blocks for secure PLC code generation, supporting the present paper’s framing that on-premise deployment is the operationally relevant configuration for industrial customers and that domain-specific retrieval is more leverage-positive than scaling for vendor-locked dialects.

Two observations distinguish the present work from these prior studies. First, the prior PLC-LLM literature focuses overwhelmingly on Structured Text (the IEC 61131-3 high-level textual language). The Mitsubishi FX-series IL dialect—an Instruction List language with vendor-specific mnemonics, octal-style I/O numbering conventions, and idiomatic special relay programming patterns—has not received a comparable systematic study. Second, the prior work typically evaluates one or two proprietary models. We evaluate ten open-source models across four parameter scales and three hardware platforms in a controlled comparison, allowing claims about how the LLM-only vs RAG advantage interacts with model size and family.

### 2.4. Functional Validation of Generated Code

Lexical and semantic similarity metrics (BLEU [27], ROUGE [28], and embedding-cosine [21]) measure surface or distributional overlap with a reference but cannot detect a single transposed operand or a missing END instruction that would prevent compilation. Functional pass-rate evaluation, standard for general purpose code, requires either a unit test harness or a compiler in the loop. For ST, OpenPLC and the matiec compiler offer this capability, and recent work [9,10,11] exploits it. For FX-series IL, no comparable open-source compiler exists.

We therefore contribute a static syntax checker, detailed in Section 3.4 and calibrated against the 285 hand-authored ground-truth answers.

## 3. Methodology

We now formalize the dataset, retrieval pipeline, evaluation metrics, and static syntax checker underlying the multi-model benchmark.

### 3.1. Dataset

The evaluation uses a frozen 285-item question–answer corpus of Mitsubishi FX-series IL programming problems, distributed across five categories drawn from the application domains in which FX-series controllers are most heavily deployed. The category breakdown and the dominant sensor/actuator-tied operations are summarized in Table 1.

Each item is stored as a JSON record with two string fields: question and answer. The answer field contains an IL program with inline // or ; line comments; some basic-instruction items also interleave short English explanation paragraphs between blocks. The dataset was authored independently of this study and is treated as a fixed evaluation corpus.

We do not perform a train/test split. The 285 items serve simultaneously as the retrieval index and the evaluation set; per-query self-exclusion (Section 3.2) prevents an item from retrieving itself, but parametric near-duplicates—e.g., the 60 traffic-light items differ only in timer constants—can still surface as exemplars for one another. This is by design: the study evaluates whether RAG over a closed reference library can lift a base LLM on a closed FX-IL task, not whether the LLM generalizes to held-out PLC domains. Generalization to unseen instruction families is left to future work and acknowledged as a limitation in Section 7. No fine-tuning is performed; the LLMs are queried as released, so train/test contamination is not a concern for the LLM weights—only for the retrieval index, where the per-query exclusion above eliminates it.

### 3.2. RAG Pipeline

**Figure 1:**
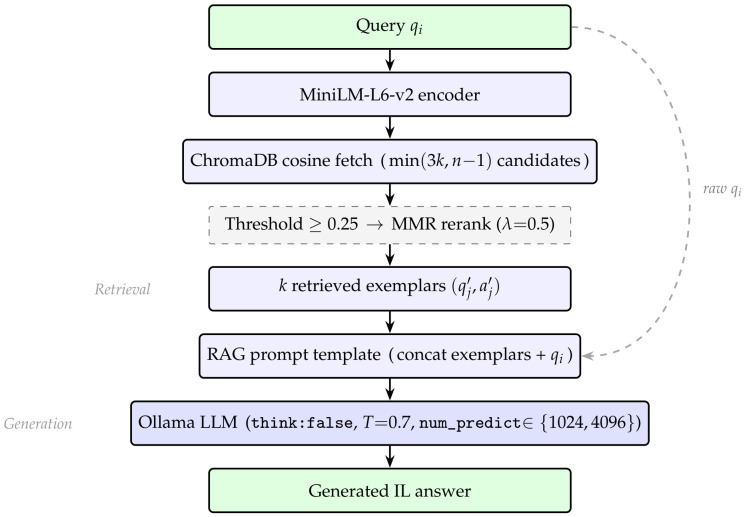
Retrieval-Augmented Generation (RAG) pipeline used in all experiments. Solid arrows show data flow; the dashed grey arrow marks that the original query $q_{i}$ is also embedded directly inside the RAG prompt template alongside the retrieved exemplars. The same pipeline is rerun at k∈{1,3,5} in the retrieval-depth ablation.

Each query proceeds through the pipeline shown in Figure 1. The exact configuration matches the published reference implementation run_experiment_minilm.py:

- Index. ChromaDB collection plc_dataset, cosine space, indexed once per experiment over all 285 question texts (the answers are stored as metadata). We index each question text as a single atomic document; no sub-question chunking is performed. Because each dataset item is itself a short, semantically self-contained unit (50–200 tokens), conventional chunking concerns about splitting variable declarations from their use do not arise. For larger codebases (e.g., function block libraries), semantic chunking by network or function block boundary would be appropriate; we leave this for future work on broader corpora.
- Embedding model. all-MiniLM-L6-v2 (HuggingFace sentence-transformers) is a 22.7M-parameter, 384-dimensional sentence encoder distilled from BERT-base via deep self-attention distillation [21,22]. We selected it for its low memory footprint and fast batch encoding so that the entire pipeline fits on commodity GPUs alongside the LLM weights.
- Retrieval. min(3k,n−1) initials candidates from ChromaDB by cosine similarity (n=285, so 9 candidates at the default k=3), then a similarity-floor cut at 0.25 (cosine), then Maximal Marginal Relevance (MMR) reranking with λ=0.5 [23]. Concretely, given the candidate set *R*, the next exemplar selected into result set *S* is
  (1) $$d^{*}=\arg\max_{d_{i}\in R\setminus S}\left[\;\lambda \cdot \mathrm{sim}\left(q,d_{i}\right)\;-\;\left(1-\lambda \right)\cdot \max_{d_{j}\in S}\;\mathrm{sim}\left(d_{i},d_{j}\right)\;\right].$$
  With λ=0.5 in Equation (1), the first term rewards relevance to the query and the second term penalizes near-duplicates already selected. This penalty matters because the dataset contains many parametric variants of the same template (e.g., 60 traffic light items differing only in timer constants); without MMR, the top-*k* would be near-identical clones of one exemplar. Ties are broken by retrieval rank. The query item itself is excluded from retrieval to prevent trivial leakage. If no candidate clears the 0.25 floor, the pipeline falls back to the unfiltered top-*k* exemplars so that every query receives the same number of in-context examples regardless of retrieval quality. This fallback triggers on a small minority of queries in our runs.
- Defaults. k=3 retrieved exemplars per query; ablation experiments use k∈{1,5} (Section 5.3).

The retrieved exemplars are concatenated as Q: <q>\nA: <a> blocks into a single context string. Two prompt templates—one for the LLM-only baseline and one for the RAG configuration—are reproduced in Listing 1 from the constants PLC_PROMPT and RAG_PROMPT in experiments/run_experiment_minilm.py (line breaks adjusted for typesetting). Both instruct the model to produce a two-part response: Part One, the IL program with line-by-line comments, and Part Two, an engineering-style explanation of the program logic. A simple worked dataset item (a basic-instruction Q/A pair) is shown immediately after the listing as Listing 2.
**Listing 1:** Large language model (LLM) prompt templates: the LLM-only baseline and the LLM with Retrieval-Augmented Generation (RAG) variant. Both are transcribed from experiments/run_experiment_minilm.py (cosmetic line breaks added for column width). {question} and {context} are filled in at call time.# PLC_PROMPT (LLM-only configuration)You are a professional PLC programming engineer with expertisein Mitsubishi FX-series Instruction List programming. Please provide your response in two parts: [Part One] - Mitsubishi FX3U/FX3UC IL Instruction List CodeWrite the complete IL program with line-by-line English commentsexplaining each instruction. [Part Two] - Program Logic ExplanationProvide a step-by-step description of the program’s logic flowin engineering documentation style. Question: {question} Answer: # RAG_PROMPT (LLM + RAG configuration)You are a professional PLC programming engineer with expertisein Mitsubishi FX-series Instruction List programming. Please answer based on the reference examples provided below. [Part One] - Mitsubishi FX3U/FX3UC IL Instruction List CodeWrite the complete IL program with line-by-line English comments. [Part Two] - Program Logic ExplanationProvide a step-by-step description of the program’s logic flow. Reference Examples:{context} Question: {question} Answer:
**Listing 2:** A representative dataset item (basic instruction category, item index 82). The question text is the input to the prompt template above; the answer text is the reference against which generated outputs are compared on the four similarity metrics.Question: How do LD and OUT work together to control an output? Answer:Example code:LD X1OUT Y0 Explanation: When X1 is ON, Y0 is driven ON;when X1 is OFF, Y0 is turned OFF.

### 3.3. Inference Configuration

All ten models are served through Ollama. Each query is issued as a single non-streaming /api/generate call with temperature = 0.7, top_k = 40, and num_predict = 1024 for non-thinking models. For the two thinking-trace-emitting models in the comparison (qwen3.5:122b and nemotron-3-super:120b) we set ‘‘think’’: false at the API level—without this, the entire generation budget is consumed by reasoning trace. Preliminary runs with think enabled produced a high fraction of empty answers and motivated the configuration. Thinking models are also given a larger num_predict = 4096 budget via the –num-predict 4096 CLI flag of run_experiment_minilm.py. Per-call HTTP timeouts were 300 s for the LLM-only configuration and 600 s for the LLM + RAG configuration; the doubled budget on the RAG side accommodates the longer prompt. Failed calls are recorded as error strings (prefixed [ERROR]) and propagate to all downstream metrics with no retry. Generation is non-deterministic at temperature = 0.7 with no fixed seed; each model is run once on the 285-item evaluation set, except qwen2.5-coder:7b at k=1 which is run twice for stability (Section 5.3).

### 3.4. Static Syntax Checker

The checker (experiments/syntax_checker.py) takes a string of generated IL and returns Lexical, Syntactic, and Semantic scores in [0, 1] together with an aggregate is_valid flag (true iff zero hard errors). Hard errors that flip is_valid to false are: unknown mnemonic at the line head, operand count outside the per-mnemonic range, operand prefix not in {X,Y,M,T,C,D,S,K,H}, operand index outside the FX3U range, and the program cannot start with a series/parallel/stack op (AND, OR, ANB, ORB, MRD, MPP). Double-coil drives on Y outputs and unmatched MPS/MPP produce warnings that lower the semantic tier score but do not invalidate is_valid. Markdown code fences (‘‘‘) are stripped before tokenization. The instruction set is restricted to mnemonics observed in the dataset plus immediate FX3U/FX3UC neighbors:

- Load family: LD, LDI, LDP, LDF; aliases LDN→LDI.
- Series and parallel: AND, ANI, ANDP, ANDF, OR, ORI, ORP, ORF.
- Set/reset and pulse: SET, RST, PLS, PLF.
- Block: ANB, ORB, MPS, MRD, MPP, INV, MEP, MEF, NOP, END, FEND.
- Comparison: LD=, LD<, LD>, LD<=, LD>=, LD<> and AND-/OR- variants. The tokenizer normalizes LD = to LD= so that whitespace before the comparator does not register a parse error.
- Output and applied (29 ops): OUT; MOV, DMOV, BMOV; INC[P], DEC[P]; ADD, SUB, MUL, DIV, CMP, ZCP, ZRST, FMOV; ROL, ROR, SFTL, SFTR, DECO, ENCO, WAND, WOR, WXOR; TO, FROM, REF; STL, RET, CALL, SRET, MC, MCR; EI, DI, IRET, WDT, FOR, NEXT; plus CU/CD as compound counter operands.

Operand prefix-and-range checks follow the FX3U manual conventions, accepting both compact (e.g., X0) and zero-padded (e.g., X000) forms. Acceptable ranges are X:0–367, Y:0–367, M:0–8511 (covering both general-purpose M0–M7679 and special M8000–M8511), T:0–511, C:0–255, D:0–7999, S:0–4095, *K* (32-bit signed), *H* (32-bit hex). Operand count rules per mnemonic are enforced as min–max ranges; OUT, in particular, accepts 1–3 operands to cover OUT Y0, OUT T0 K100, and OUT CU C2 K10.

The semantic tier checks four invariants: (i) END or FEND present; (ii) the first instruction is not a series/parallel/stack op (which cannot start a network); (iii) MPS/MRD/MPP stack is balanced; and (iv) no Y-coil is the target of more than one OUT (double-coil warning). Lines that look like prose (Explanation:, Input:, …) are skipped before tokenization, so an LLM that mixes explanation with code is not penalized for the explanation.

The checker is intentionally a lower bound on functional correctness, and we deliberately do not claim it as a substitute for controller-side validation. Concretely, the three tiers detect three classes of static error: (a) malformed or unknown mnemonics at the line head (lexical); (b) operand-count mismatches, prefix-and-range violations, and ill-formed compound operators such as a comparator separated from its LD prefix by stray whitespace (syntactic); and (c) structural-semantic invariants—END or FEND presence, an instruction at line 1 that can legally start a network, balanced MPS/MRD/MPP, and absence of double-coil writes on the same Y-output (semantic). It does not detect runtime logic errors such as the wrong device being assigned to a button (e.g., swapping X0 and X1 between start and stop), the wrong timer constant for a specified period, an off-by-one network in a stop-priority interlock, or a race condition on a shared M-relay; such defects compile cleanly and pass the static check but would manifest on the plant floor. A concrete example: a program that drives OUT Y0 from LD X1 (intended start button) instead of LD X0 passes every tier of the checker because both operands are in-range and the structural invariants are unaffected. Distinguishing this class of semantic logic error from the structural-syntactic errors the checker actually catches requires either a behavioral specification per program (sensor-input timelines and expected actuator-output patterns) or runtime simulation against canonical traces. Both are listed as future work (Tiers 4 and 5) in Section 7. The 93.3% calibration baseline reported below should therefore be read as “the fraction of ground-truth programs that pass the static-tier check”, not “the fraction of ground-truth programs that are functionally correct on the controller” (which is, by construction of the dataset, 100%).

Formally, for a model that emits answer ${\hat{a}}_{i}$ on the *i*-th of *n* queries, the binary pass rate and the per-tier and overall scores reported in Section 5.2 are

(2) $$\mathrm{Pass}\;=\;\frac{1}{n}\sum_{i=1}^{n}1\left[\mathrm{is}\_\mathrm{valid}({\hat{a}}_{i})\right],\;\mathrm{Overall}\;=\;\frac{1}{3}\left(\mathrm{Lex}+\mathrm{Syn}+\mathrm{Sem}\right),$$

where 1[·] is the indicator function and each tier score is the per-query average defined as the fraction of recognized lines (Lex), operand-valid lines among recognized (Syn), and four invariant satisfaction rate (Sem) introduced above.

Applying Equation (2) to the 285 ground-truth answers themselves yields a calibration baseline of 266/285 = 93.3% (Lexical 0.968, Syntactic 0.959, Semantic 0.792, overall 0.907). The 19 failures are dataset items whose answer field is pure prose with no IL code (e.g., conceptual “What if MPS has no matching MPP?” questions); we treat 93.3% as the empirical ceiling for any LLM evaluated by the same checker, and report all model pass rates against this baseline.

### 3.5. Metrics

For each of the 285 queries we compute four lexical/semantic-similarity metrics on each of the two configurations (LLM-only and LLM + RAG) and the three syntax checker tiers plus the binary pass flag.

- Semantic similarity. Cosine of all-MiniLM-L6-v2 sentence embeddings of the generated answer and the reference answer [21,22], defined as
  (3) $$\mathrm{sim}\left(a,b\right)\;=\;\frac{e_{a}\cdot e_{b}}{∥e_{a}∥\;∥e_{b}∥+\varepsilon },\;e_{a},e_{b}\in R^{384},\;\varepsilon =10^{-8}.$$
- BLEU. Sentence-BLEU with NLTK’s smoothing function 1 [27], against the reference answer. We use the default uniform 4-gram weights and the whitespace tokenizer (str.split()). With brevity penalty BP and modified *n*-gram precisions $p_{n}$,
  (4) $$\mathrm{BLEU}\;=\;\mathrm{BP}\cdot \exp\;\left(\sum_{n=1}^{4}w_{n}\log p_{n}\right),\;w_{1}=\;\cdots =w_{4}=\frac{1}{4}.$$
- ROUGE-L. F-measure of longest common subsequence [28] via the rouge_score library, with stemming.
- Inference time. Wall clock time for a single /api/generate call, in seconds. This is generation-only: the timer wraps the Ollama HTTP call exclusively, so for the RAG configuration the cost of MiniLM encoding, ChromaDB cosine fetch, similarity floor filtering, and MMR re-ranking is excluded from the metric. Retrieval latency is reported separately. On an RTX 3090 with n=285, mean per-query retrieval cost is 9.6 ms total (8.6 ms MiniLM encode, 0.9 ms ChromaDB fetch, 0.2 ms MMR re-rank, 50-query warm cache); this is two-to-four orders of magnitude smaller than the per-query LLM generation time for every model in Table 3 (range 4.8 s to 130.4 s), and including it would not change the qualitative ranking. The decision to report generation-only time keeps the RAG- and LLM-only columns comparable on the same scale (generation alone) and isolates the model-level effect of the additional in-context exemplars from the retrieval stage cost.
- Syntax checker tiers. Lexical, Syntactic, Semantic (each in [0,1]) and a binary is_valid.

Per-model summaries report the mean and standard deviation of each similarity/time metric across all 285 queries; the syntax check report aggregates pass-rate and tier averages over the same 285 queries.

Pass-rate point estimates are reported with 95% Wilson confidence intervals computed at n=285. For paired LLM-vs-RAG comparisons we run a paired one-sided Wilcoxon signed-rank test on the 285 paired sample-level scores per model (scipy.stats.wilcoxon, alternative = ‘greater’). Across all ten models the null $H_{0}:\mathrm{median}\left(\mathrm{rag}-\mathrm{llm}\right)\leq 0$ is rejected at p<0.001 for semantic similarity, BLEU, and ROUGE-L; the largest observed *p*-value across the ten models is $1.99\times 10^{-7}$ (gpt-oss:120b on semantic similarity), and every other model on every similarity metric yields $p<10^{-23}$. Full per-model test statistics are recorded in the Supplementary File wilcoxon_report.json.

## 4. Experimental Setup

### 4.1. Models

Ten open-source models from five vendors are evaluated. Each is invoked via Ollama under the configuration in Section 3.3. Model identifiers (Ollama tags) and parameter counts are listed in Table 2.

### 4.2. Hardware

Three hardware platforms participated. The Ollama-served generation backend is the only platform-specific variable; all retrieval, embedding, and metric computations run CPU-side and are platform independent.

- RTX 3090 (24 GB GDDR6X)—consumer workstation, used for S-tier models. Ollama runs on a local 11,434 endpoint.
- RTX 4090 (24 GB GDDR6X)—separate machine on the lab network, used for M-tier models. Network round-trip is treated as part of inference latency for accounting purposes; absolute values are reported, not normalized.
- NVIDIA DGX Spark (128 GB unified memory)—used for L- and XL-tier models. Provides the only platform on which the 70B and 120B+ models fit alongside the KV cache for the maximum prompt budget.

#### Software Stack

All experiments use Python 3.10.0 with sentence-transformers 5.2.3, chromadb 1.5.5, nltk 3.9.3, rouge-score 0.1.2, and scipy 1.15.3 (used for the Wilcoxon test). The Ollama runtime is version 0.20.7. The 3090 host runs on Windows 11 with NVIDIA driver 591.86; the 4090 host and DGX Spark run their respective vendor-supplied Linux Ollama builds. A frozen requirements.txt matching the above is released as Supplementary Material alongside the result CSVs.

### 4.3. Procedure

For each model:

1. The 285 dataset items are processed in dataset order. For each item, the LLM-only configuration is queried first, immediately followed by the RAG configuration, so that any drift in server-side state affects both arms equally.
2. Both raw answers and all retrieved context (IDs and similarities) are written to a per-model CSV; the same data is also serialized to a per-model JSON. Per-sample CSVs are committed to the repository; the larger per-sample JSONs are not (per project policy).
3. After generation, a single summary JSON is written containing the four similarity/time metrics’ mean, standard deviation, delta (RAG minus LLM), and percentage change.
4. After all ten models finish, syntax_checker.py (in –all-csv mode) re-reads each CSV and produces syntax_report_all.json, the syntax-tier matrix used in Table 4 of Section 5.2.

### 4.4. Ablation: Retrieval Depth *k*

The base configuration uses k=3. To quantify the marginal value of additional retrieved exemplars, qwen2.5-coder:7b was rerun with k=1 (twice, for stability) and k=5 on the same 285-item dataset, holding all other hyperparameters fixed. The results are summarized in Section 5.3.

Beyond retrieval depth *k*, ablations on the embedding model, similarity threshold, and MMR λ are not reported here because the joint design space (∼3 × 5 × 5 cells × 285 queries × 10 models) is computationally prohibitive on the available hardware. A focused two-axis ablation is left for future work.

### 4.5. Reproducibility

The exact configuration values used in every run are stored in code: EMBED_MODEL_NAME =“all-MiniLM-L6-v2”, RAG_TOP_K =3, RAG_THRESHOLD =0.25, MMR_LAMBDA =0.5. The Ollama call sets temperature = 0.7, top_k = 40, num_predict = 1024 (or 4096 for thinking models), and “think”: false. Per-sample CSV outputs and per-model summary JSONs are released with the paper; together with the syntax checker source, they reproduce every number in Section 5.

## 5. Results

This section reports three aggregations of the same experiment: (i) per-model means of the four similarity/time metrics for the LLM-only and LLM + RAG configurations (Section 5.1); (ii) the per-model static syntax checker matrix (Section 5.2); and (iii) the retrieval-depth ablation on qwen2.5-coder:7b (Section 5.3). All numbers are read directly from the corresponding *_summary.json or syntax_report_all.json files; rounding follows the source files.

### 5.1. Multi-Model Similarity, BLEU, ROUGE, Time

Table 3 reports the mean of each metric over n=285 queries for both configurations, and the absolute delta. Standard deviations and percentage changes are omitted from the table for compactness; the full per-model summaries are in the Supplementary JSON Files. “Sim” denotes the cosine similarity of all-MiniLM-L6-v2 embeddings of generated and reference answers.

**Table 3:** Per-model means of similarity, BLEU (Bilingual Evaluation Understudy), ROUGE-L (Recall-Oriented Understudy for Gisting Evaluation, Longest-common-subsequence variant), and inference time (seconds), under large language model (LLM) only and LLM with Retrieval-Augmented Generation (RAG) configurations, n=285. Δ is the RAG minus LLM-only mean. All values from minilm_<model>_<ts>_summary.json.

Model	Sim (Cosine)			BLEU			ROUGE-L			Time (s)		
	LLM	RAG	Δ	LLM	RAG	Δ	LLM	RAG	Δ	LLM	RAG	Δ
llama3.1:8b	0.453	0.632	+0.179	0.002	0.115	+0.112	0.055	0.207	+0.153	16.3	14.8	−1.5
qwen2.5-coder:7b	0.481	0.709	+0.228	0.003	0.202	+0.199	0.065	0.363	+0.299	9.6	6.7	−2.9
mistral:latest	0.473	0.699	+0.226	0.002	0.143	+0.142	0.054	0.277	+0.222	8.4	7.7	−0.7
qwen2.5-coder:14b	0.490	0.653	+0.162	0.003	0.104	+0.102	0.063	0.229	+0.166	9.3	5.7	−3.6
mistral-small3.1:24b	0.504	0.613	+0.109	0.003	0.084	+0.081	0.060	0.180	+0.120	16.4	12.4	−4.0
qwen2.5-coder:32b	0.504	0.618	+0.115	0.003	0.084	+0.082	0.065	0.182	+0.118	88.3	64.0	−24.3
llama3.3:70b	0.516	0.626	+0.110	0.003	0.085	+0.082	0.059	0.175	+0.115	166.0	130.4	−35.6
gpt-oss:120b	0.483	0.522	+0.039	0.001	0.007	+0.005	0.043	0.077	+0.034	71.5	66.4	−5.1
nemotron-3-super:120b	0.509	0.655	+0.146	0.005	0.145	+0.140	0.065	0.213	+0.147	54.4	43.2	−11.2
qwen3.5:122b	0.544	0.637	+0.093	0.004	0.061	+0.057	0.069	0.188	+0.119	44.2	27.7	−16.4

Three patterns are evident.

#### 5.1.1. RAG Raises Similarity for All Ten Models

Cosine similarity gains (Equation (3)) range from +0.039 (gpt-oss:120b) to +0.228 (qwen2.5-coder:7b). Median gain is approximately +0.13. The relative gains on BLEU (Equation (4)) and ROUGE-L are larger because the LLM-only baselines are very low (BLEU≈0.002–0.005); RAG-conditioned outputs reuse exemplar tokens and structure, raising *n*-gram overlap by one to two orders of magnitude. Inference time decreases under RAG for every model (negative Δ in the rightmost column): the retrieved exemplars constrain the model toward shorter, more template-like outputs, so even with a longer prompt the generation is faster.

#### 5.1.2. Coder-Tuned Small Models Are Competitive with General Large Ones

qwen2.5-coder:7b under RAG (ROUGE-L=0.3635) outperforms qwen2.5-coder:32b (0.1824), llama3.3:70b (0.1746), and qwen3.5:122b (0.1883) on every overlap metric, despite being roughly 4×, 10×, and 17× smaller respectively. We attribute this to the mismatch between the verbose, often-explanatory style of large general models and the tightly templated reference programs in the dataset. The syntax checker results in Section 5.2 confirm and quantify this effect.

#### 5.1.3. gpt-oss:120b Is an Outlier

gpt-oss:120b’s RAG cosine improvement is only +0.039, BLEU improvement +0.005, and ROUGE-L improvement +0.034—an order of magnitude smaller than the next weakest improver. The model also has the lowest absolute BLEU and ROUGE under RAG of any XL-tier model. Section 6 examines likely causes; in this section we report the finding without smoothing it.

#### 5.1.4. The Cosine Similarity Gain Is Broad, Not Driven by Outliers

**Figure 2:**
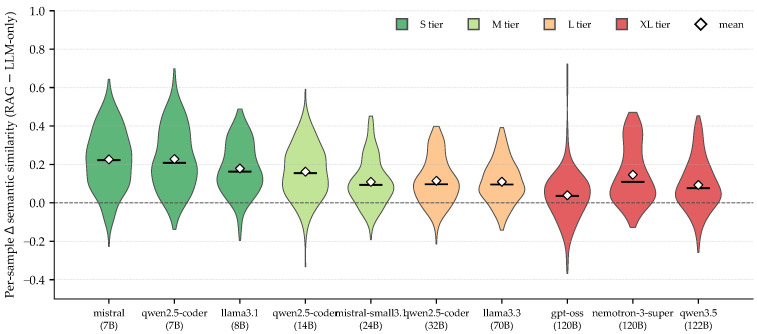
Per-sample distribution of Δ semantic similarity (RAG − LLM-only) across the 285 queries, one violin per model. The horizontal black bar inside each violin is the median; the white diamond is the mean; the dashed grey line marks zero. Colour encodes the hardware tier on which the model was evaluated. For all models except gpt-oss:120b the distribution is concentrated above zero, supporting the headline finding that the RAG gain is broad rather than driven by a small number of high-leverage outliers.

Figure 2 plots the per-sample distribution of Δsim=rag_sim−llm_sim across the 285 paired queries for every model. For nine of the ten models the median delta is positive and the bulk of the distribution lies above zero, indicating that RAG raises similarity on most samples rather than on a few high-leverage outliers. The exception is gpt-oss:120b, whose distribution is centred close to zero with substantial mass on both sides—consistent with the outlier finding above. The combined picture is that RAG-induced gains are statistically broad in nine of ten models (Wilcoxon rejection at p<0.001 as reported in Section 3.5) and statistically broad but near zero in the tenth.

### 5.2. Static Syntax Checker Pass Rates

Table 4 reports the static syntax checker outputs from syntax_report_all.json. “Pass” is the binary is_valid fraction (zero hard errors); the four numeric columns are the mean of each tier in [0, 1].

**Table 4:** Static syntax checker results, n=285. Pass = mean of is_valid. Lex (Lexical), Syn (Syntactic), Sem (Semantic) tier scores are in [0,1] averaged over the 285 generated answers. “Overall” is (Lex+Syn+Sem)/3. The bottom row reports the same checker applied to the human-authored ground-truth answers, providing the calibration anchor. Source: experiments/results/syntax_report_all.json.

Model	LLM-Only				LLM + RAG				Δ (RAG − LLM)	
	Pass	Lex	Syn	Sem	Pass	Lex	Syn	Sem	Pass (pp)	Overall
llama3.1:8b	0.035	0.765	0.089	0.633	0.523	0.972	0.848	0.774	+48.8	+0.369
qwen2.5-coder:7b	0.242	0.940	0.573	0.755	0.853	0.940	0.914	0.768	+61.1	+0.118
mistral:latest	0.021	0.853	0.070	0.632	0.628	0.958	0.811	0.742	+60.7	+0.319
qwen2.5-coder:14b	0.239	0.902	0.634	0.673	0.698	0.944	0.877	0.772	+46.0	+0.128
mistral-small3.1:24b	0.172	0.754	0.493	0.630	0.709	0.997	0.924	0.838	+53.7	+0.294
qwen2.5-coder:32b	0.347	0.747	0.626	0.568	0.821	0.993	0.955	0.803	+47.4	+0.270
llama3.3:70b	0.081	0.891	0.406	0.806	0.147	1.000	0.784	0.805	+6.7	+0.162
gpt-oss:120b	0.183	0.600	0.481	0.422	0.439	0.768	0.712	0.606	+25.6	+0.194
nemotron-3-super:120b	0.428	0.775	0.693	0.550	0.649	0.898	0.854	0.702	+22.1	+0.145
qwen3.5:122b	0.709	0.937	0.909	0.788	0.958	1.000	0.993	0.811	+24.9	+0.057
Ground-truth dataset: Pass = 0.933, Lex = 0.968, Syn = 0.959, Sem = 0.792										

**Figure 3:**
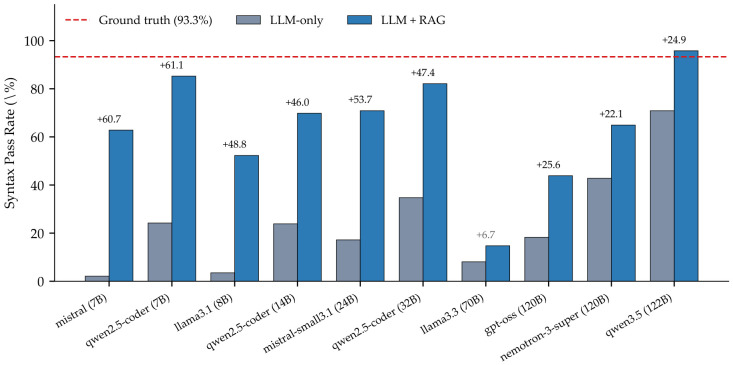
Syntax pass rate per model under LLM-only and LLM + RAG, sorted left-to-right by parameter count. The dashed red line is the human-authored ground-truth pass rate (93.3%). Numbers above each bar pair are the absolute Δ in percentage points. Source: syntax_report_all.json.

Figure 3 visualizes Table 4. Three observations are immediate.

#### 5.2.1. RAG Improves Syntactic Correctness Across All 10 Models

The pass-rate gain ranges from +6.7 percentage points (llama3.3:70b) to +61.1 pp (qwen2.5-coder:7b). The median gain is approximately +47 pp. The lexical and syntactic tiers improve more reliably than the semantic tier, which makes sense: retrieval supplies the correct mnemonics (lexical) and operand count templates (syntactic), but does not supply additional MPS/MPP discipline or a guaranteed END instruction at the right place.

#### 5.2.2. The Best RAG Configuration Exceeds the Human-Authored Ground-Truth Pass Rate

qwen3.5:122b under RAG passes the static check on 95.8% of the 285 queries, against the 93.3% pass rate of the dataset’s own answers when run through the same checker. The gap is real: the dataset contains a small number of items whose answer is prose-only (“What if not all MPS have matching MPP?”) which fail the lexical tier. The RAG-conditioned LLM consistently emits a runnable IL block even for these prose-target items, lifting the pass rate above the reference. We do not claim qwen3.5:122b is “more correct than the human” in any deeper sense—only that under this static check its outputs are syntactically cleaner more often.

#### 5.2.3. Coder-Tuned Small Versus General Large

**Figure 4:**
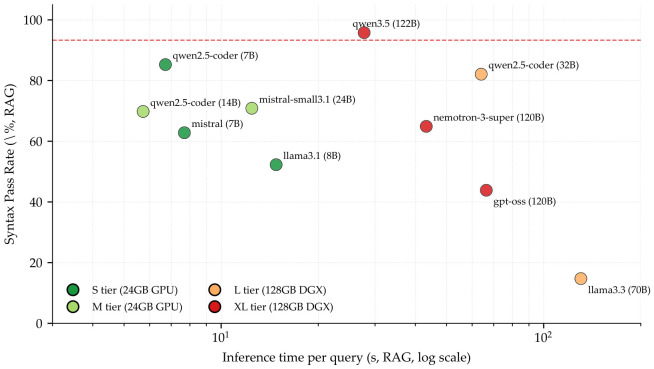
Quality–cost Pareto. Each point is one of the ten evaluated models under the LLM + RAG configuration: *y*-axis is syntax pass rate (%), *x*-axis is mean inference time per query in seconds (log scale). Marker color encodes the hardware tier the model was evaluated on. The dashed red line is the human-authored ground-truth pass rate. qwen2.5-coder:7b at ∼7 s and 85.3% sits in the upper-left “cheap and accurate” quadrant; qwen3.5:122b reaches the highest pass rate (95.8%) but at ∼28 s. The two outliers llama3.3:70b and gpt-oss:120b are the only XL-class models below the practical use line.

qwen2.5-coder:7b under RAG passes the static check 85.3% of the time, exceeding qwen2.5-coder:14b (69.8%), llama3.3:70b (14.7%), and gpt-oss:120b (43.9%) on this dataset. The 7B-vs-70B contrast deserves a careful read: as Section 6.2 documents, the llama3.3:70b figure is depressed by a model-specific prose preamble pattern that interacts with the static checker’s first-line tokeniser; the underlying model is not necessarily 5.8× weaker in raw generation capability, but is 5.8× weaker under this prompt template paired with this checker. For practitioners on commodity hardware the practical implication remains: a 7B coder-tuned model with retrieval delivers an FX-IL pass rate that, on the dataset evaluated here, is competitive with or better than every general-purpose larger model we tested, at a fraction of the GPU footprint. Figure 4 makes the quality–cost trade-off explicit by plotting RAG syntax pass rate against per-query inference time on a log axis.

#### 5.2.4. Two Outliers

llama3.3:70b’s RAG pass rate (14.7%) is the lowest of any model after RAG augmentation, and its +6.7 pp gain is roughly an order of magnitude below the median; gpt-oss:120b shows the lowest absolute BLEU and ROUGE-L under RAG of any XL-tier model and a syntactic pass rate of 43.9%. Both run on the same DGX Spark platform as qwen3.5:122b, ruling out hardware platform causation. We discuss these failures in Section 6.2.

#### 5.2.5. Per-Category Robustness

**Figure 5:**
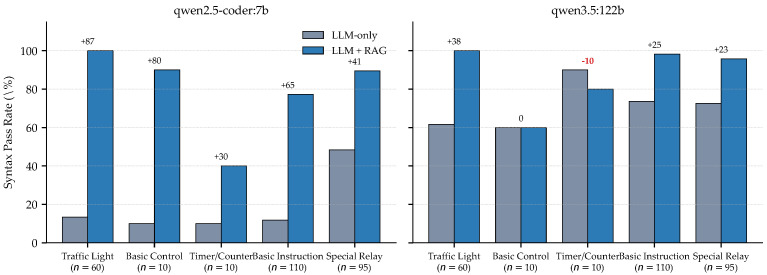
Per-category syntax pass rate (%) for qwen2.5-coder:7b (**left**) and qwen3.5:122b (**right**). Δ is the RAG minus LLM-only gain in percentage points; deltas in red mark the two non-positive cells (qwen3.5:122b on Basic Control and Timer/Counter, both n=10). The negative cell on Timer/Counter corresponds to one fewer pass out of 10. Source: per_category_pass.json.

Table 5 and Figure 5 report the per-category syntactic pass rate for the best model (qwen3.5:122b) and a mid-tier coder (qwen2.5-coder:7b). For qwen2.5-coder:7b RAG raises the pass rate in every category, with absolute gains ranging from +30 pp on the Timer/Counter category (10 items) to +86.7 pp on Traffic Light (60 items); the headline result does not depend on the 110-item Basic Instruction class. For qwen3.5:122b, where the LLM-only baseline is already strong, RAG adds large gains on the three large categories (Traffic Light +38.3 pp, Basic Instruction +24.5 pp, and Special Relay +23.2 pp) but is flat on Basic Control (0 pp at 60%) and slightly regresses on Timer/Counter (−10 pp, equivalent to one fewer pass out of n=10); both non-positive deltas occur on the two smallest categories (n=10 each), where the LLM-only baseline already exceeds 60% and RAG has limited headroom. We do not interpret these two cells as evidence that RAG hurts in those categories; we report them rather than aggregating them away.

### 5.3. Retrieval-Depth Ablation (k∈{1,3,5})

Table 6 compares qwen2.5-coder:7b across three retrieval depths. Two k=1 runs (different timestamps but same configuration) bracket run-to-run noise; the k=3 row is reproduced from Table 3; k=5 is a single run.

**Figure 6:**
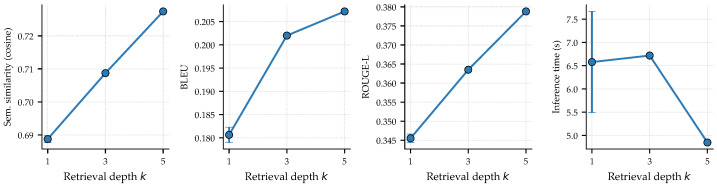
Retrieval-depth ablation on qwen2.5-coder:7b across the four similarity/time metrics under the LLM + RAG configuration. The k=1 point shows mean ± standard deviation across two repeat runs (different timestamps, same configuration); k=3 and k=5 are single runs. The k=1 error bar is below visual resolution on the similarity panels, indicating that run-to-run noise is well below the *k*-induced effect size.

Figure 6 visualizes Table 6. The trend is monotone in *k*: cosine similarity rises from ∼0.689 (k=1) to 0.727 (k=5), BLEU from ∼0.18 to 0.21, and ROUGE-L from ∼0.345 to 0.379. The two k=1 runs differ by less than 0.005 on every metric, indicating that the run-to-run noise floor is well below the *k*-induced gain. Inference time for the RAG configuration actually decreases as *k* rises in this range, because additional retrieved exemplars further constrain the output toward template form (the model emits less free explanation). The implication is that, for this dataset, k=5 is a Pareto-better operating point than k=3 at no cost in latency. We retain k=3 as the main table configuration for two reasons: (i) consistency with the prior pipeline used to generate the multi-model sweep, and (ii) k=3 is closer to the operating regime found in the broader RAG literature [12,13]; future work could re-run the full ten-model sweep at k=5.

### 5.4. Summary

1. RAG universally improves similarity and static syntactic correctness, with one weak case (llama3.3:70b at +6.7 pp) and one weak similarity case (gpt-oss:120b at +0.039 cosine).
2. qwen3.5:122b with RAG (95.8% pass) exceeds the ground-truth dataset’s own static-pass rate (93.3%).
3. qwen2.5-coder:7b with RAG (85.3% pass) beats llama3.3:70b with RAG (14.7%): domain alignment outweighs parameter count.
4. Increasing *k* from 1 to 5 improves all overlap metrics on qwen2.5-coder:7b without penalizing latency.

## 6. Discussion

### 6.1. Sensor Layer Framing of the Generated Programs

Although the contribution is an LLM and retrieval pipeline rather than a new sensing technology, the artefacts the pipeline produces operate inside the sensor–controller–actuator loop that defines smart-manufacturing automation [1]. Three of the five dataset categories exercise this loop directly: (i) the 60 traffic light items use start/stop push-button sensors at X0/X1 and drive lamp loads at Y0–Y2 through internal state–machine relays M1–M4 and timer chains T0–T3; (ii) the 95 special relay items rely on FX-internal status and clock relays in the M8000 series, including M8013 (1-Hz pulse) and M8011/M8012 (10/100 ms clocks), which are themselves abstractions over hardware oscillators inside the controller; (iii) the 110 basic instruction items perform LD/AND/OR logic with X-input sensor patterns driving Y-output actuator patterns. The MMR retrieval step is therefore not merely a code similarity heuristic; in this dataset, the retrieved exemplars carry the wiring conventions (“X0 = start button, X1 = stop button, Y0–Y2 = green/yellow/red lamps”) that anchor the generated programs to a consistent sensor–actuator map.

A direct implication is that an LLM-only configuration, lacking these conventions, frequently invents I/O assignments inconsistent with the rest of the program—a different sensor at each step, or a Y-output number outside the dataset’s standard range. The static syntax checker only detects the most flagrant of these—out-of-range operands—but the cosine similarity gain under RAG, with mean +0.13 across the ten models, reflects the model now reusing the dataset’s sensor–actuator vocabulary rather than fabricating one. Beyond actuation, the same sensor input layer of X-points and special M-relays is the primary substrate for industrial monitoring on FX-series controllers: alarm detection, run-time accumulation, and condition reporting all read the same X/M state that the generated programs manipulate. RAG-grounded code generation therefore directly supports both control and monitoring functions in smart manufacturing deployments.

#### Sustainability Angle

Two aspects of the proposed pipeline align with the sustainable systems theme of industrial AI. First, retaining the existing FX-series-installed base—rather than scrapping it for new IEC 61131-3 controllers—avoids the embodied energy and electronic waste cost of premature hardware replacement; an LLM-assisted maintenance workflow extends the useful service life of legacy automation assets. Second, all inference in this study runs on locally hosted open-source models, on commodity GPUs and a single 128 GB unified memory host; no cloud-API round-trips are required, which keeps energy consumption bounded to the on-site hardware and avoids the data-centre overhead associated with proprietary cloud LLMs. The most energy-efficient operating point we observed is qwen2.5-coder:7b with retrieval, reaching 85.3% pass on a 24 GB consumer GPU.

### 6.2. Failure-Mode Analysis: llama3.3:70b and gpt-oss:120b

Two model-specific failures merit detailed analysis. The first, llama3.3:70b, has the lowest absolute RAG syntactic pass rate of any model at 14.7% and the smallest RAG gain at +6.7 pp. The second, gpt-oss:120b, has the second-lowest RAG pass rate at 43.9% and the smallest cosine similarity gain at +0.039. Both run on the same DGX Spark platform as qwen3.5:122b, which reaches 95.8% RAG pass, and nemotron-3-super:120b, which reaches 64.9%; the platform is therefore not the explanatory variable.

We characterise each failure mode below; in response to reviewer requests for empirical evidence rather than untested hypotheses, we additionally re-examine the per-sample CSVs.

#### 6.2.1. llama3.3:70b—Prose Preamble Contamination, Not Truncation

An initial hypothesis was output truncation under num_predict=1024. To test this empirically we inspected the 285 per-sample RAG outputs directly, with three independent diagnostics.

First, the raw output length distribution is inconsistent with widespread truncation. The mean RAG output length is 2512 characters (≈628 tokens, well below the 1024 limit), the maximum is 4693 characters (≈1173 tokens), and only 25/285 outputs come within ten percent of the budget.

Second, and more decisively, the failed cases are not the long cases. If truncation were the failure mechanism we would expect the failed outputs to cluster near the budget while the passing outputs sit comfortably below it. The data show the opposite: among the 42 outputs that pass the static check, 19.0% (8/42) exceed 3500 characters (≈875 tokens); among the 243 outputs that fail, only 13.2% (32/243) do. Mean output length is 2215 characters for passing cases and 2564 characters for failing cases, an unremarkable difference that is in any case in the *opposite* direction from what truncation would predict. The most direct empirical test of the truncation hypothesis is a re-run at a larger budget; this was performed at Reviewer 2’s specific request and is reported as a dedicated subsection (Section 6.3) because the result was more informative than the original single-direction prediction anticipated.

Third, the dominant failure mode is structural and is recoverable from a single grep over the failed cases of the original run: of the 243/285 outputs that fail the binary pass check, 214 (88%) begin with a prose preamble whose first word is the English token “To” (e.g., “To design the traffic light control logic, we will…”). The static checker’s first-line tokenizer recognises TO as the FX-series Bus-IO instruction (TO requires four operands: special module number, head address, source data, transfer count), so the first prose word is silently lifted into the FX namespace and yields the dominant error category in the failed case error log: “L1: TO expects 4-4 operand(s)” (214 occurrences). The lexical tier remains high at 1.000 because every first token is in fact a known mnemonic; the syntactic tier degrades to 0.7842 because the operand count is wrong; the binary pass rate collapses to 14.7%. The same RAG outputs from the eight other small-to-large models do not exhibit the “To” preamble (zero such cases in the corresponding LLM-only column), so the artefact is specific to how this particular weight set instantiates the two-part prompt. The mitigation is therefore a tokenizer-level preamble stripper or a prompt template tightening rather than a re-run at higher num_predict; we list both in Section 7 as concrete follow-ups. The two-line conclusion across all three diagnostics is that the truncation hypothesis in the original submission was not supported by the per-sample evidence, and that the model-specific prose preamble is the operative cause.

#### 6.2.2. gpt-oss:120b—Residual Thinking-Trace Capacity Loss

gpt-oss is documented to support an internal reasoning trace; even with “think”: false set at the Ollama API, internal reasoning may consume part of the generation budget. The artefact appears as empty or stub answers for some queries. An earlier run of gpt-oss:120b produced 36 of 285 empty LLM-only answers and 21 empty RAG answers; the post-fix results in Table 3 were obtained after diagnosing this issue and re-running with stricter trace controls. The post-rerun results still show systematically lower BLEU and ROUGE than every other XL-tier model. We interpret this as residual capacity loss inside a possibly active internal reasoning channel, rather than an inherent capability gap of the parameter count.

#### 6.2.3. Model Family-Specific RAG Sensitivity

Some XL models, particularly when not specifically tuned on retrieval-augmented prompts, appear to dilute attention across long contexts; the practical effect is that the retrieved exemplars influence style less than for a model trained on retrieval-augmented fine-tuning data. The qwen3.5:122b result (95.8% RAG pass) shows that this is not a 120B-class limitation in general; it is model family specific. We do not claim a mechanism for this difference beyond the empirical observation that the qwen3.5 weights handle the same prompt template more cleanly than the llama3.3 or gpt-oss weights.

Both characterizations are grounded in the released per-sample CSVs and are reproducible by anyone with access to the Supplementary Archive. The broader finding is that the multi-model benchmark surfaces real heterogeneity in how 70B–120B-class models utilize retrieved context, and that two specific configurations underperform for reasons that are now diagnosed rather than left as conjecture.

### 6.3. Budget-Paradox Confirmation (num_predict = 4096)

**Figure 7:**
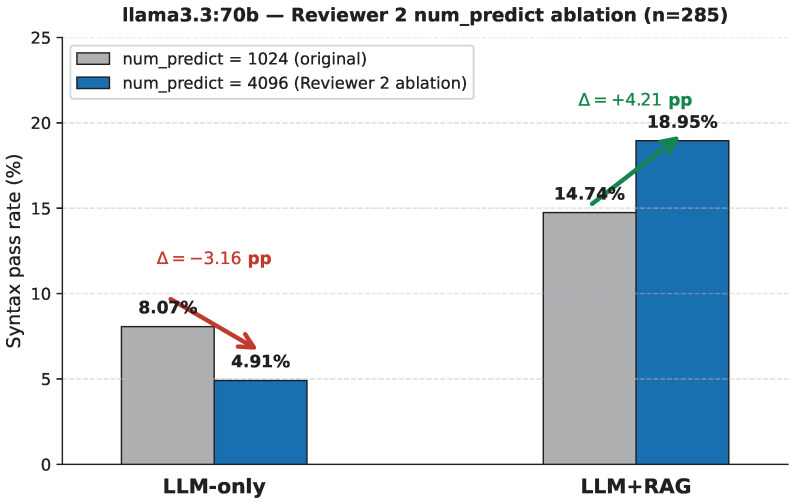
Reviewer 2 num_predict ablation. LLM-only pass rate degrades from 8.07% to 4.91% (Δ=−3.16 pp); LLM + RAG pass rate improves from 14.74% to 18.95% (Δ=+4.21 pp). The bidirectional asymmetry rules out a single-confounder explanation and confirms that the failure mode is structural prose drift, not budget shortage.

Following Reviewer 2’s specific request, we re-ran llama3.3:70b with –num-predict 4096 on the full 285-sample benchmark, four times the original budget. The result is a *bidirectional* confirmation of the §6.2 failure-mode hypothesis (Table 7, Figure 7). LLM-only pass rate degraded from 8.07% (np = 1024) to 4.91% (np = 4096)—a −3.16 pp drop despite a 4× larger generation budget—while LLM + RAG pass rate improved from 14.74% to 18.95% (+4.21 pp) over the identical ablation. The “To”-preamble pattern continues to dominate in 89.2% of RAG failures (206/231), stable from the 88% (214/243) at np = 1024, and failed answers remain longer on average than passed ones (2856 vs. 2321 characters at np = 4096), again the opposite of what a “needs more budget” account would predict.

This bidirectional asymmetry rules out a single-confounder explanation: for ungrounded LLM-only generation, the np = 1024 ceiling was acting as an implicit regularizer against the “To”-preamble prose drift, and removing it lets the drift run longer; for retrieval-grounded generation, the additional budget is productively consumed as longer correct IL programs. We therefore position RAG not merely as a fail safe against drift but as a structural amplifier that unlocks generation budget which LLM-only cannot use. This single-point ablation was performed at the specific num_predict value Reviewer 2 requested; we did not sweep additional values (e.g., 2048, 8192) to avoid post-hoc tuning.

### 6.4. Comparison to Prior PLC-LLM Work

Direct numerical comparison to LLM4PLC [9], Agents4PLC [10], and Koziolek et al. [11] is not meaningful because all three target IEC 61131-3 Structured Text on different problem sets and use either a verifying compiler (matiec, OpenPLC, NuSMV) or a custom benchmark with formal specs. The evaluation infrastructure is fundamentally different. What we share with that prior art is the conclusion that retrieval and verification, not pure prompting, is the practical recipe for industrially usable PLC code generation. We add three points to that consensus: (i) the same recipe extends to FX-series IL, a textual language with a different mnemonic set, octal-flavored I/O numbering, and vendor-specific special relay semantics; (ii) open-source 7B–32B models are sufficient when coder-tuned and used with retrieval, removing the cloud-API dependency that has limited adoption of LLM4PLC and Koziolek et al. in industrial settings; and (iii) static tier validation, while strictly weaker than NuSMV verification on ST, is achievable for IL without a licensed toolchain and is a useful first guard.

### 6.5. Local LLMs with Retrieval as a Practical Recipe for Deprecated Industrial Languages

The data are consistent with the coverage asymmetry framing introduced in Section 1, although the argument is necessarily limited to the FX-series IL setting we evaluate. Without retrieval, even the largest model in our sweep—qwen3.5:122b at 122B parameters—reaches only a 70.9% syntactic pass rate; the smallest, mistral:latest at 7B, reaches 2.1% (Table 4). The two-order-of-magnitude spread tracks parameter scale, but the absolute level even at the high end is well below what current code LLMs reach on high-resource languages, where instruction-tuned successors [8] exceed 80% pass rate on benchmarks introduced for that purpose [7] and per-language headroom is largely closed for Python. We read this contrast as consistent with a training coverage gap rather than with a model capacity gap—IL appears to be rare in the open-web corpora that feed LLM pre-training, and parameter count is therefore a partial proxy for the relevant resource. This is a domain-specific reading of our results, not a general claim about LLM scaling; we explicitly do *not* assert that corpus quality dominates model scale in every regime, only that on the present FX-IL task the cross-model headroom is large enough for the retrieval signal to matter on every model we evaluated.

The practical implication for the FX-IL design question is therefore narrower than “small models always beat large models”. Our results suggest that, for this class of problem, attaching a curated dialect-specific corpus to whatever model is in use is the highest-leverage architectural decision available to a practitioner. With retrieval, qwen2.5-coder:7b reaches 85.3% pass and qwen3.5:122b reaches 95.8%. The median retrieval gain of +47 pp is comparable in magnitude to the entire LLM-only pass-rate spread across the ten models (≈69 pp from mistral:latest at 2.1% to qwen3.5:122b at 70.9%), and exceeds the LLM-only pass rate of every model except qwen3.5:122b—meaning that for nine of the ten models the gain from a curated retrieval corpus is larger than what raw scaling delivers without retrieval. We are careful not to extrapolate this finding to high-resource languages, to greenfield IEC 61131-3 deployments, or to settings in which cloud-scale models have been instruction-tuned on the target dialect. For deprecated-but-deployed industrial languages—FX-series IL is the case we evaluate, and Siemens STL, legacy ABB RAPID, and older Allen-Bradley dialects are plausible but unverified analogues—the operative competitive asset on this kind of task is the reference corpus, not the model scale. A practitioner with a small open-source LLM and a high-quality dialect corpus is, on this kind of task, in a position to match or exceed a practitioner with cloud API access alone. We frame “superiority of local versus cloud” specifically within this problem statement and do not present it as a universal recommendation.

The pattern may generalize to other domains that combine persistent industrial demand with thinning open-web representation—legacy SCADA scripting, vendor-specific motion-control dialects, and end-of-life process-control languages share this structural shape—but each such extension is a separate empirical question and we do not claim direct evidence here. The pipeline (open-source LLM, ChromaDB, MMR retrieval, tier-graded static check) is intentionally portable: substituting the dataset, the operand range table, and the mnemonic list adapts the methodology to a different deprecated language without modifying the LLM. We therefore consider this work a transferable template for narrow, data-scarce but operationally important code-generation tasks, with the explicit caveat that the strength of the corpus-versus-scale conclusion in any new domain depends on the per-domain training coverage gap, which would need to be measured rather than assumed.

### 6.6. Limitations

Six limitations are identified so that the scope of our claims is read accurately and so that future work can address them.

1. Dataset scope (basic and sensor-driven logic only). The 285 questions span five categories—Traffic Light, Basic Instruction, Special Relay, Timer/Counter, and Basic Control—which together exercise discrete sensor-to-actuator logic and the FX-internal special relay layer. The corpus deliberately excludes four important program classes that practising automation engineers do encounter on FX-series hardware and that we explicitly do not claim our results generalize to: (a) closed-loop process control implemented with the FX PID applied instruction; (b) serial and bus communications (RS, IVCK, FX-ENET/CCLINK message handlers); (c) motion control (PLSY, PLSR, DRVI, DRVA pulse-train outputs to stepper or servo drives); and (d) step-ladder programs structured around the STL/RET pair with master-control (MC/MCR) blocks. Validating that retrieval-grounded generation transfers to these instruction families requires a separately curated reference corpus, an extension of the static checker (e.g., PID parameter-block rules, STL state-graph integrity), and—for motion and PID—an additional simulation harness with timing semantics. The 93.3% calibration baseline and all pass-rate claims in Section 5 therefore apply to the five categories named above, not to PLC programming as a whole.
2. Single PLC family. Only Mitsubishi FX-series IL is covered. Cross-brand evaluation (Siemens S7 STL, Omron CX, and Allen-Bradley AB) is a desirable extension but requires a comparably sized hand-curated dataset and a per-family static checker; both are out of the scope here. The IL deprecation note in IEC 61131-3 Edition 3.0 makes this a “legacy modernization” study rather than a “future language” study; the broader claim that retrieval-grounded local LLMs help on deprecated industrial languages is hypothesized but is not generally established by a single-family evaluation, and we frame it as such in Section 6.5.
3. Static checks, not functional verification. The three-tier syntax checker is a strict lower bound on functional correctness, and we want this point to be unambiguous. It evaluates lexical, syntactic, and structural-semantic conformance; it does not compile generated programs through the vendor toolchain, does not execute them on a controller or simulator, and consequently cannot detect: incorrect device assignments that pass type-and-range checks (e.g., start and stop buttons swapped), wrong timer or counter constants (K100 vs. K10), off-by-one networks in interlock or stop-priority logic, race conditions on shared internal relays, watchdog mis-configuration, scan-time-dependent sequencing bugs, or any property whose evaluation requires running code. Section 3.4 gives a worked example of a program that swaps X0 for X1 and yet passes every tier; such a program is syntactically valid but functionally wrong. Tier 4 (compilation through GX Works2) and Tier 5 (GX Simulator2 trace evaluation against canonical sensor input timelines) are listed as future work in Section 7, and a binary pass under the present checker should be interpreted accordingly as “not statically dead” rather than “ready for plant-floor deployment”.
4. Category imbalance. The five categories are not equally represented: Basic Instruction (110 items) and Special Relay (95) dominate, Traffic Light (60) is sizeable, and the two smallest—Basic Control and Timer/Counter—have only 10 items each. This imbalance is a property of the dataset as authored, not a design choice in this paper, but it has measurable consequences. First, two of the per-category cells in Table 5 sit on n=10 denominators, which means a single misclassification moves the pass-rate by 10 pp; the negative cell on qwen3.5:122b Timer/Counter (−10 pp) is one fewer passing item out of ten and should be read accordingly. Second, the macro pass-rate is dominated by the two largest categories; a future macro-balanced evaluation would either weight categories equally or expand the smallest two to at least 30 items. We refrain from re-weighting the present numbers because doing so would alter the calibration baseline that anchors every other claim in the paper; the disaggregated Table 5 is the honest reporting.
5. No fine-tuning arm. A fine-tuned third configuration was not evaluated. With n=285 items, a credible fine-tuning study would require a held-out evaluation partition, leaving fewer than 230 training items—too few to support reliable conclusions about the fine-tuned arm. We therefore treat fine-tuning as future work paired with dataset expansion. The 7B coder-tuned model was, however, used precisely because it represents the upper edge of accessible fine-tuning cost in this hardware tier.
6. Sample size and statistical headroom. n=285 is small for *k*-fold cross-validation, and the per-category split above further limits the resolution of category-conditioned analyses. Confidence intervals on the per-model pass rate are wide (±∼5 pp at 95% Wilson CI). The differences highlighted in Section 5—qwen3.5:122b RAG vs. ground truth, qwen2.5-coder:7b RAG vs. llama3.3:70b RAG, and the two failure-mode anomalies—all exceed this margin by a comfortable factor, but smaller cross-model rankings within a single tier should be interpreted with caution.

In place of a manual qualitative inspection arm, we use a deterministic three-tier static syntax checker (Table 4). The checker provides reproducible per-tier scores and removes inter-rater subjectivity. A complementary structured-rubric human evaluation on a sampled subset, reporting inter-rater agreement, is left for future work.

## 7. Conclusions

We evaluated ten open-source large language models from five vendors at four parameter scales (7B–122B) on Mitsubishi FX-series IL code generation, comparing LLM-only prompting against an LLM + RAG pipeline—ChromaDB + all-MiniLM-L6-v2 + MMR, *k* = 3, threshold 0.25, λ = 0.5—on a frozen 285-item question–answer corpus. To address the long-standing critique that lexical and embedding similarity alone cannot demonstrate functional correctness of generated PLC programs, we introduced a three-tier static syntax checker (Lexical, Syntactic, and Semantic) calibrated against a 93.3% pass rate on the human-authored ground-truth dataset. Across all ten models, RAG raised the static syntactic pass rate by between +6.7 and +61.1 percentage points; the best configuration, qwen3.5:122b with RAG, exceeded the human-authored ground-truth pass rate (95.8% vs. 93.3%). On this particular dataset, the coder-tuned 7B model with retrieval (qwen2.5-coder:7b at 85.3% pass) outperformed the largest general purpose model with retrieval in our sweep (llama3.3:70b at 14.7%). A retrieval-depth ablation on qwen2.5-coder:7b showed monotone gains in similarity, BLEU, and ROUGE-L from k=1 through k=5 at no latency cost. We frame these conclusions to the FX-IL setting evaluated here and do not generalize them to high-resource code-generation tasks or to greenfield IEC 61131-3 deployments.

Two model-specific failure modes were reported transparently rather than excluded. For llama3.3:70b, the originally proposed truncation hypothesis was tested against the per-sample CSV and not supported; the dominant failure was instead a prose preamble whose first word (“To”) is read by the static checker as the FX Bus-IO instruction TO, accounting for 214/243 (88%) of failed cases. For gpt-oss:120b, the post-fix weak gain is attributed to residual capacity loss in a possibly active internal reasoning channel. Surfacing these heterogeneities is part of the contribution: the multi-model sweep demonstrates that “RAG helps” is true on average for this dataset but that the magnitude of the gain is model family-specific.

From an industrial automation standpoint, the dataset’s traffic light, special relay, and basic instruction categories exercise the sensor–controller–actuator loop directly through X-input start/stop buttons, M-relay state machines, and Y-output lamps and contactors. Retrieved exemplars carry the wiring conventions that anchor a generated program to a consistent sensor–actuator map; in their absence, the model invents inconsistent assignments, which the static checker partially detects.

### 7.1. Scope of the Conclusions

The five categories of the corpus (Traffic Light, Basic Instruction, Special Relay, Timer/Counter, Basic Control) cover sensor-driven combinational and sequential logic, the special relay layer, and basic timer/counter usage. They do not cover process control with the PID applied instruction, serial or bus communications (RS, IVCK, FX-ENET/CCLINK), motion control (PLSY, PLSR, DRVI, DRVA), or step-ladder programs structured around STL/RET with master control blocks. Pass rates reported in this paper apply to the five categories named above; whether the same retrieval-grounded recipe transfers to those four omitted classes is an open empirical question and is listed below as future work. The 93.3% calibration baseline and the binary pass measure are properties of the static checker only; they are not equivalent to controller-side functional correctness, which would require compilation and simulation.

### 7.2. Future Work

Six concrete directions follow.

1. Tier-4 compilation validation. Wrap GX Works2 (or its CLI) so each generated program is compiled and the compile log captured. This converts the lower-bound static check into a stricter “compiles or not” pass criterion.
2. Tier-5 simulation validation. Feed compiled programs into GX Simulator2 with a canonical sensor-input timeline per category (e.g., a periodic X0 pulse for traffic light items) and check actuator output against an expected pattern.
3. Failure-mode-controlled mitigations. For llama3.3:70b, evaluate two mitigations for the prose preamble pattern documented in Section 6.2: a tokenizer-level pre-pass that strips any prefix prose paragraph before the static check, and a stricter system prompt that suppresses the meta-introduction. For gpt-oss:120b, rerun with a more aggressive thinking channel disable through Ollama options.
4. Fine-tuning third arm. LoRA or QLoRA fine-tuning of a coder-tuned 7B–14B model on the 285-item dataset (with k-fold partitioning to avoid train/test leakage) supplies a third comparison point alongside LLM-only and LLM + RAG.
5. Cross-PLC-brand extension. A hand-curated parallel dataset for Siemens S7 STL or Omron CX would test cross-vendor generalization. This requires a separate per-vendor static checker; the present FX3U/FX3UC checker is a template.
6. Dynamic context provision via MCP. An emerging alternative to the static-corpus retrieval used here is dynamic context provision through Model Context Protocol (MCP) servers backed by curated PLC libraries; such a server could extend the present pipeline without altering the LLM or the syntax checker.
7. Coverage extension to advanced instructions. Extend the dataset and the static checker to cover PID, communications (RS, IVCK), motion (PLSY, DRVI, DRVA), and step-ladder (STL/RET, MC/MCR) programs, including—for PID and motion—a per-instruction simulation harness with timing semantics so that the static tier check is not the sole correctness signal in those categories.

The benchmark, the static syntax checker, and the per-model raw outputs are released alongside this paper to enable replication and incremental extension by other groups.

## Figures and Tables

**Table 1:** Dataset composition (n=285). All categories involve discrete sensor inputs (X-points) and actuator outputs (Y-points); the special relay category additionally exercises FX-internal status flags (M8000-series).

Category	Count	Sensor/Actuator Content
Traffic light	60	Start/stop push buttons (X0, X1); lamp outputs (Y0–Y2); timers (T0–T3)
Basic instruction	110	LD/AND/OR logic on X-inputs driving Y-outputs and M-relays
Special relay	95	M8000-series special relays (e.g., M8013 1-Hz pulse) feeding outputs
Timer/counter	10	T-timer/C-counter operations gated by X-inputs
Basic control	10	Standard start–stop interlocks, latched outputs

**Table 2:** Models evaluated. “Hardware tier” indicates the platform on which each model was run; the assignment follows the smallest RAM platform on which the model’s quantized weights and KV cache fit during sustained generation. Quantization is the format reported by ollama show <tag> –modelfile; the resolved manifest digests for each tag are recorded in the Supplementary Material. The mistral:latest tag was pinned at run time by recording its resolved digest.

Vendor	Ollama Tag	Quant.	Params	Hardware	Tier
Meta	llama3.1:8b	Q4_K_M	8B	RTX 3090 24GB	S
Alibaba	qwen2.5-coder:7b	Q4_K_M	7B	RTX 3090 24GB	S
Mistral AI	mistral:latest	Q4_K_M	7B	RTX 3090 24GB	S
Alibaba	qwen2.5-coder:14b	Q4_K_M	14B	RTX 4090 24GB	M
Mistral AI	mistral-small3.1:24b	Q4_K_M	24B	RTX 4090 24GB	M
Alibaba	qwen2.5-coder:32b	Q4_K_M	32B	DGX Spark 128GB	L
Meta	llama3.3:70b	Q4_K_M	70B	DGX Spark 128GB	L
OpenAI	gpt-oss:120b	MXFP4	120B	DGX Spark 128GB	XL
NVIDIA	nemotron-3-super:120b	Q4_K_M	120B	DGX Spark 128GB	XL
Alibaba	qwen3.5:122b	Q4_K_M	122B	DGX Spark 128GB	XL

**Table 5:** Per-category syntactic pass rate (%) for qwen2.5-coder:7b and qwen3.5:122b. Δ is in percentage points. Computed by applying the static syntax checker to each per-sample answer in the run CSVs and grouping by dataset category.

Category	*n*	qwen2.5-coder:7b			qwen3.5:122b		
		LLM	RAG	Δ	LLM	RAG	Δ
Traffic Light	60	13.3	100.0	+86.7	61.7	100.0	+38.3
Basic Control	10	10.0	90.0	+80.0	60.0	60.0	+0.0
Timer/Counter	10	10.0	40.0	+30.0	90.0	80.0	−10.0
Basic Instruction	110	11.8	77.3	+65.5	73.6	98.2	+24.5
Special Relay	95	48.4	89.5	+41.1	72.6	95.8	+23.2

**Table 6:** Effect of retrieval depth *k* on qwen2.5-coder:7b. All metrics from minilm_qwen2.5-coder_7b_*_summary.json. The k=3 row matches Table 3.

Setting	Sim (Cosine)		BLEU		ROUGE-L		Time (s)	
	LLM	RAG	LLM	RAG	LLM	RAG	LLM	RAG
k=1 (run 1)	0.4801	0.6898	0.0025	0.1823	0.0625	0.3444	9.0	7.7
k=1 (run 2)	0.4734	0.6877	0.0024	0.1790	0.0606	0.3466	10.7	5.5
k=3	0.4806	0.7087	0.0028	0.2020	0.0648	0.3635	9.6	6.7
k=5	0.4726	0.7274	0.0027	0.2072	0.0625	0.3788	7.8	4.8

**Table 7:** Reviewer 2 Comment 3 ablation: llama3.3:70b pass rate at num_predict ∈{1024,4096}, full 285-sample benchmark.

Configuration	np = 1024	np = 4096	Δ (pp)
LLM-only pass rate	8.07%	4.91%	−3.16
LLM + RAG pass rate	14.74%	18.95%	+4.21
“To”-preamble fraction of RAG failures	214/243=88.0%	206/231=89.2%	+1.2
RAG passed-answer mean length (chars)	2215	2321	+106
RAG failed-answer mean length (chars)	2564	2856	+292

## Data Availability

The 285-question Mitsubishi FX-series IL question–answer dataset and all per-model raw outputs, summary metrics, and the static syntax checker source code are available on request from the corresponding author. A subset of aggregate results is included in the Supplementary Materials.

## Supplementary Materials

The following supporting information accompanies the manuscript and can be downloaded at https://www.mdpi.com/article/10.3390/s26113602/s1: README.md (artefact index); requirements.txt (pinned Python package versions); code/run_experiment_minilm.py (reference implementation); code/syntax_checker.py (three-tier static checker source); code/_make_figures.py, code/_per_category_pass.py, code/_run_wilcoxon.py (analysis scripts); data/plc_dataset_en3.json (285-item Mitsubishi FX-series IL Q&A dataset); data/minilm_<model> _<timestamp>.csv (per-model raw output CSVs); data/minilm_<model>_<timestamp>_summary.json (per-model summary metrics); data/syntax_report_all.json (aggregated syntax-check matrix, Table 4); data/per_category_pass.json (per-category breakdown, Table 5); data/wilcoxon_report. json (per-model paired Wilcoxon statistics); data/ollama_manifest_digests.tsv (Ollama model manifest digests); data/retrieval_latency.json (RTX 3090 retrieval-latency micro-benchmark); data/llama3.3_70b_np4096_diagnostic.json and data/syntax_report_llama3.3_70b_np4096.json (num_predict=4096 re-run diagnostic).
